# Advocating for improvements to building codes for the population’s health

**DOI:** 10.17269/s41997-019-00191-7

**Published:** 2019-02-14

**Authors:** Nancy Edwards, James Chauvin, Rosanne Blanchet

**Affiliations:** 1 School of Nursing, University of Ottawa, Room E125, 200 Lees Avenue, Ottawa, ON K1S 5L5 Canada; 2 Gatineau, Canada; 3 Edmonton Clinic Health Academy, University of Alberta, 4-398, Mailbox #54, 11405 87 Avenue, Edmonton, Alberta T6G 2P5 Canada

**Keywords:** Falls, Injuries, Construction codes, Public health advocacy, Public policy, Chutes, Traumatismes, Codes de construction, Défense de la santé publique, Politique publique

## Abstract

Construction codes are a major component of building codes. They provide normative standards by which buildings are designed, built, altered, inspected, and assessed. Persistently high, fall-related injury rates on stairs and in bathrooms indicate that public health advocacy is needed to enhance the passive protection of these codes. Targets and strategies for code improvement advocacy by public health professionals, organizations, and associations are discussed. Approaches pertinent to describing the problem, proposing solutions, and framing the message are considered. Attention is given to issues that may be particularly challenging for advocates. These include the need to address minimum standards, tackling gaps in injury-related surveillance data that may be used by the building industry to rebut proposed code changes, describing how construction code changes align with other progressive legal tools that shape our built environments, and considering which sector pays and which sector benefits from code improvements. Ergonomic and epidemiologic evidence indicates that construction code improvements can reduce falls and fall-related injuries. Public health advocates have an important role to play in strengthening these codes.

## Résumé

Les codes de construction constituent un élément majeur des codes du bâtiment. On y trouve les critères normatifs de conception, de construction, de modification, d’inspection et d’évaluation des bâtiments. Les taux constamment élevés de blessures liés aux chutes accidentelles dans les escaliers et les salles de bain indiquent que des campagnes de santé publique sont nécessaires pour améliorer la protection passive offerte par ces codes. Notre commentaire traite des cibles et des stratégies d’éventuelles campagnes d’amélioration des codes de construction à mener par les professionnels, les organismes et les associations de la santé publique. Des démarches pertinentes pour décrire le problème, proposer des solutions et formuler le message sont examinées. Une attention particulière est accordée aux questions les plus épineuses pour les défenseurs de la santé publique : les révisions nécessaires aux normes minimales, les lacunes dans les données de surveillance des traumatismes qui peuvent être mises à profit par le secteur du bâtiment pour réfuter les modifications proposées, l’harmonisation entre les modifications aux codes de construction et les autres outils juridiques progressistes qui donnent forme à nos milieux bâtis, et l’étude des secteurs qui paient pour les améliorations aux codes et de ceux qui en bénéficient. Selon les données ergonomiques et épidémiologiques, les améliorations aux codes de construction peuvent réduire les chutes accidentelles et les traumatismes liés aux chutes. Les défenseurs de la santé publique ont un rôle important à jouer dans le renforcement de ces codes.

Construction codes (building, fire, energy, plumbing, and electrical) are critical drivers of the built environment. They provide normative standards by which buildings are designed, built, altered, inspected, and assessed. Regulated at national and provincial/state levels, and enforced at provincial/state and local levels, these technically detailed codes offer passive protection for the population. Public health proponents have successfully advocated for improvements to fire codes, yielding better sprinkler systems, the use of fire walls and improved routes of egress, all contributing to reductions in burns and fatalities (U.S. Fire Administration, 2018; Licht, 2005). However, public health has paid less attention to building codes.

While a number of health issues can be tackled through building codes (World Health Organization, 2018), this commentary focuses on preventing falls and injuries involving stairs and bathrooms. Stair- and bathtub/shower-related falls ranked first and eighth, respectively, among the top 10 consumer products in the United States involved in non-fatal injury costs (Consumer Product Safety Commission, 2017). For 2009–2010, the annual incidence of non-fatal injuries related to stairs exceeded 1.23 million persons, while for bathtubs/showers, it was estimated to be nearly 263,000 people. The total estimated annual direct and indirect costs for stair-related non-fatal injuries exceeded US $92 billion over that same period; costs associated with bathtub/shower-related non-fatal injuries exceeded US $19 billion (Lawrence et al., 2015). Canadian data suggest a similar pattern. Falls are the leading cause of injury-related deaths and hospitalization in Canada (Parachute, 2015). Falls on stairs were the most frequent cause of falls deaths in 2010. Many provincial health ministries identify fall prevention as a priority given the annual economic burden of falls in Canada, estimated at $8.7 billion (Parachute, 2015).

While causes of falls are often considered multifactorial, ergonomic and epidemiological studies indicate that certain features of stairs and bathrooms (e.g., dimensional inconsistencies and lack of handrails on stairs, absence of grab bars in bathtubs) directly contribute to the risk of falls and related injuries in these locations (Jackson & Cohen, 1995; Stevens et al., 2011; Joint Task Group on Step Dimensions in Dwelling Units, 2013). Randomized controlled trials of home modifications indicate that falls can be prevented through structural improvements (Keall et al., 2015). A population health approach to reduce fall-related injuries on stairs and in bathrooms involves providing better passive protection through building codes (Edwards 2017). Strong advocacy by public health professionals and associations is needed to generate momentum for this improved passive protection. Based in part on our (NE & JC) experience on national code committees in Canada, we suggest several promising approaches for public health advocacy.

## Public health advocacy strategies

Advocates have a good starting point for success, since Canada’s national building codes include health and safety as prime objectives (Canadian Commission on Building and Fire Codes/National Research Council of Canada, 2015). Input on codes may be provided through a request for a code change; support for or opposition to a proposed code change under discussion by technical review committees; and written submissions made during time-limited, public consultations on proposed code changes. Position statements outlining the need for code changes can be prepared for endorsement by public health associations, prompting their advocacy on specific code issues. Successful public health advocacy entails describing the problem and proposing solutions. Framing messages is important for both.

## Describing the problem

The principle of minimum standards (rather than best practices) is used as a basis for consensus decisions about code changes. Also called minimum requirements and minimum level of performance, these standards have their roots in the professional codes of engineers and architects. They define the lowest technical requirement (in the case of prescriptive codes) or the lower-most acceptable level of risk or performance (in the case of objective and performance-based codes).

It is critical for public health advocates to gain an understanding of how the term “minimum standards” is used by members of code-related technical review committees they want to influence. Speaking to members of technical committees, discussing arguments used by proponents or adversaries of code changes and reviewing code change documents that are in the public domain may provide insights on how minimum standards have previously been defined and used by a specific technical committee.

In describing the need for a code change, advocates are making the case for a shift in the acceptable minimum standard. To do so, it is essential to use injury data to demonstrate that the current level of risk, stipulated by the minimum standard, is unacceptable. Ergonomic studies are an important source of data to build cases, but they demonstrate what happens in laboratory rather than real-world settings. Nevertheless, ergonomic studies have been used to show what tread width is needed for the safer navigation of stairs (see example in Textbox 1) and what grab bars are needed for safely entering and exiting a bathtub.

**Textbox 1**

**Table:** 

Stair-related fall injuries are a major cause of deaths and injuries in both Canada and the US. The public health community in Canada advocated to amend the code for residential dwelling stairs from the then-existing run length dimension of a minimum of 210 mm to a minimum 280 mm (estimated to result in an 80% reduction in fall risk) and a maximum rise dimension of 180 mm. This would have brought the code for residential dwellings in line with the existing standard for public (non-residential dwelling) stairs. The thrust of the evidence presented by public health advocates was ergonomic rather than epidemiological. This was due, in part, to the lack of a robust and comprehensive national surveillance database connecting the environment (stairway characteristics) to fall-related injuries and the propensity of technical committee members to attribute falls on stairs primarily to human behaviour (distraction, drinking, etc.). Architects’ and building industry proponents’ counter-arguments cited concerns about having to change stair-manufacturing processes, discarding existing stock because it would no longer be code-compliant, and the redesign of residential dwellings due to an increased stairway footprint in increasingly small plots of land. Their argument centered on the costs associated with a projected decrease in new housing demand due to increased costs of manufacturing and installing stairways with different dimensions and implications for jobs within the building materials and construction industry. In the end, the code change agreed to was a small increase in the run length dimension (from 210 to 255 mm) and no change in the rise dimension (it remained at 200 mm). Overall, the argument for an incremental, minimalist code change carried more weight than that presented by the public health community.	

However, public health arguments for code changes could be stronger if specific parameters of the built environment were documented in comprehensive injury surveillance data, allowing the attribution of these structural dimensions to falls. Such data would provide more compelling evidence of the magnitude of health and safety improvements (or deteriorations) that could be expected through a code change in both the short and longer term. Thus, better injury surveillance data is an issue meriting the attention of public health advocates.

In Canada, injury surveillance data are collected primarily through emergency room-based reports and supplemented by longitudinal studies. Several organizations (Parachute Canada, Canadian Institutes of Health Information, Public Health Agency of Canada, provincial ministries of health) release periodic reports about the magnitude, location, and cost of falls and fall-related injuries. However, these data lack details on physical attributes of the built environment that are implicated in falls, such as the diameter of handrails, the position of bathroom grab bars, and the height of stair risers. The Canadian Hospitals Injury Reporting and Prevention Program (CHIRPP), in its current form, does not provide a code-linked data set. Even comprehensive surveillance systems of large populations, such as the US Consumer Product Safety Commission National Electronic Injury Surveillance System, lack this specificity (Stevens et al., 2011; Blazewick et al., 2018).

Advocacy for improvements in injury surveillance and related data collection systems is urgently needed. Otherwise, this evidence void will continue to be used as a counter-argument for progressive code changes.

## Proposing solutions

Codes are part of a larger set of legislative frameworks and regulations that shape our built environment. Describing how suggested code changes align with other progressive legal tools may provide the basis for strategically arguing for health- and safety-related improvements in building codes. For instance, the Americans with Disability Act of 1990 drove code changes to improve accessibility of homes and public buildings. In Canada, the Accessibility for Ontarians with Disability Act of 2005 has been successfully used to advance more accessibility-oriented building code legislation in Ontario. Similarly, the intended impact of aging-in-place policies may be used to build a case for code changes that mitigate risks for falling, such as the mandatory provision of grab bars in bathrooms. Furthermore, regulatory codes can be exceeded, providing latitude to advocate for stronger municipal regulations such as by-laws. Incremental steps towards code change can also be achieved at the local level by consortia and alliances aiming to improve accessible housing or increase visitability of homes.

When proposing (or opposing) code changes, it is important to consider which sector pays for and which sector benefits from these changes. Public health proponents must be cognizant of economic arguments (e.g., construction sector employment, housing sales, costs that get passed onto consumers) that may be used to thwart progressive code changes. For example, in the for-profit sector, a code change may have cost implications along the entire supply chain, resulting in new manufacturing processes, a shift in an industry’s competitive advantage, and a subsequent loss in employment. Stressing cost savings in the health sector that could result from building code improvements such as safer stairs and bathrooms may not be compelling to those in the housing sector (see Textbox 2). Therefore, health proponents must understand the viewpoints relevant to a range of sectors, involve economists in helping to frame arguments, and advocate for the inclusion of indirect health and social costs in code change impact analyses.

**Textbox 2**

**Table:** 

This dilemma of who pays and who benefits is illustrated in the debates in Canada, the US, and Australia relating to proposed code changes about the climbability requirements for guards used where children may be present. The railing industry in Canada has consistently and, for the most part successfully, argued against any modifications, predicating their argument on the “tiny” percentage of injuries related to guard-climbability in the available US-CPSC-NEISS statistics of annual national estimates of hospital treatments (the numerator) divided by the annual national estimates of hospital treatments for all children in the 1–1/2 to 4 age range. The industry proponents argued that the problem, based on available injury statistics, was too small to warrant regulatory codes. Furthermore, the industry cited issues related to the cost implications of changing guardrail design and off-loading existing stock, as well as limited consumer preferences for design features. The industry also argued that parental surveillance of children was a more important factor in preventing child falls from guards. The result: no code modification (Lynkowski, 2010; Pauls, 2016).	

## Conclusion

Public health needs to be at decision-making tables for construction codes. Yet, we must not enter into this process naively; contributing to the code development and review process requires a solid understanding of how to formulate compelling cases for multisectoral technical committees. Public health professionals, organizations, and associations have complementary roles to play in this domain. There is a strong imperative for this advocacy work given solid evidence indicating that construction code improvements can reduce falls and fall-related injuries.
